# Application of GPS occurrence data to understand African white‐backed vultures *Gyps africanus* spatial home range overlaps

**DOI:** 10.1002/ece3.8778

**Published:** 2022-04-02

**Authors:** Mark Zvidzai, Fadzai Michelle Zengeya, Mhosisi Masocha, Henry Ndaimani, Amon Murwira

**Affiliations:** ^1^ 37595 Department of Geography and Environmental Science University of Zimbabwe Harare Zimbabwe

**Keywords:** African white‐backed vulture, autocorrelated kernel density estimator, Bhattacharyya's coefficient, home range overlap

## Abstract

Understanding key overlap zones and habitats which are intensively shared by species in space and time is crucial as it provides vital information to inform spatial conservation with maximum benefits. The advent of high‐resolution GPS technologies associated with new analytical algorithms is revolutionizing studies underpinning species spatial and social interaction patterns within ecosystems. Here, using a robust home range estimation algorithm, the autocorrelated kernel density estimator (AKDE) equipped with an equally powerful home range overlap metric, the Bhattacharyya's coefficient (BC), we provide one of the first attempts to estimate and delineate spatial home range overlap zones for critically endangered African white‐backed vultures to inform conservation planning. Six vultures were captured in Hwange National Park using a modified cannon net system after which they were tagged and tracked with high‐resolution GPS backpacks. Overall, results suggested weaker average home range overlaps based on both the pooled data (0.38 ± 0.26), wet non‐breeding seasonal data (0.32 ± 0.23), and dry breeding season data (0.34 ± 0.28). Vultures 4, 5, and 6 consistently revealed higher home range overlaps across all the scales with values ranging between 0.60 and 0.99. Individual vultures showed consistence in space use patterns as suggested by high between‐season home range overlaps, an indication that they may be largely resident within the Hwange ecosystem. Importantly, we also demonstrate that home range overlapping geographic zones are all concentrated within the protected area of Hwange National Park. Our study provides some of the first results on African vulture home range overlaps and segregation patterns in the savanna ecosystem based on unbiased telemetry data and rigorous analytical algorithms. Such knowledge may provide vital insights for prioritizing conservation efforts of key geographic overlap zones to derive maximum conservation benefits especially when targeting wide‐ranging and critically endangered African white‐backed vultures. To this end, spatial overlap zones estimated here, although based on a small sample size, could provide a strong foundation upon which other downstream social and ecological questions can be explored further to expand our understanding on shared space use mechanisms among African vulture species.

## Graphical abstract

Understanding home range spatial overlaps is critical for the conservation of critically endangered species. Application of spatially explicit remote‐tracking technologies is providing important tools for this purpose. Home range overlaps for African white‐backed vultures are complex and need further investigation.

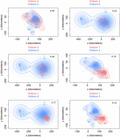

## 1 INTRODUCTION

Understanding species home range overlaps over a range of spatial and temporal scales is increasingly becoming important to wildlife ecologists as a tool for effective conservation planning. This may be because information revealing shared space use provides important insights regarding the nature and the degree of species interaction in ecosystems which has essential biological and socioecological implications (Ferrarini et al., 2021; Grammer, 2019; Hoover et al., 2020; Warning & Benedict, 2015). Besides, it also provides invaluable information regarding individual species site fidelity to specific zones, which may be surrogates for high‐quality and beneficial zones. Such zones which are attractive to large populations require strategic prioritization in terms of their conservation in order to derive maximum returns (Botha et al., 2017; Margalida & Ogada, 2018). When targeting wide‐ranging species, with extensive home ranges such as African white‐backed vultures, delineation and quantification of high‐quality zones is important in order to focus limited conservation resources to areas with the best conservation returns. Focusing conservation efforts on expansive vulture home ranges, sometimes straddling across international boundaries, is often associated with daunting challenges (Bartoń et al., 2019; Buechley et al., 2018; Efrat et al., 2020; Kark et al., 2015; Margalida & Ogada, 2018; Phipps, 2015; Runge & Tulloch, 2018). For example, the financial and human resources investments required to accomplish such a task may not be available especially for countries in the Global South (French et al., 2019; Hoover et al., 2020). Estimating and quantifying space use overlapping zones for vulture species could be the first strategic step for effective conservation planning as it provides vital information to support spatially explicit and focused conservation interventions over much smaller zones (French et al., 2019; Hoover et al., 2020). In fact, the Multi‐Species Action Plan to Conserve African‐Eurasian Vultures (Vulture MSAP), proposes the prioritization of conservation efforts targeting specific geographic zones which support vulture aggregations and interactions (Botha et al., 2017), while Xirouchakis et al. (2021) also recommended prioritizing the conservation of vulture hotspot zones as an urgent and indispensable response strategy for supporting any vulture recovery action plans (Efrat et al., 2020; Safford et al., 2019; Salewski, 2021).

Previous studies have focused on different forms of species interaction in an attempt to characterize key geographic zones which provide critical resources for species. For example, some studies have emphasized the ecological importance of species dynamic interaction, which is the degree to which pairwise moving species are interdependent (Böhm et al., 2008; Long et al., 2014; Miller, 2012). Other studies have also focused more on static interactions, which is the joint space use patterns by any two individual species without any reference to the temporal dimensions of such interactions (Fieberg & Kochanny, 2005; Kernohan et al., 2001; Millspaugh et al., 2004; Robert et al., 2012). Both these forms of interaction indeed provide important insights on how species organize themselves in ecosystems as they attempt to optimize survival and breeding success.

Species static interactions can best be understood based on segregation or overlapping space use between individuals and populations (Long et al., 2014; Warning & Benedict, 2015; Winner et al., 2018). To this end, several home range overlap metrics that quantify species static or spatial interactions have been tested and reviewed (Fieberg & Kochanny, 2005; Millspaugh et al., 2004; Robert et al., 2012). Among the metrics most frequently applied are percent area overlap (HR), volume of intersection (VI), Bhattacharyya's affinity (BA), utilization distribution overlap index (UDOI), and Spearman's correlation coefficient. Previous reviews of these metrics have demonstrated that overlap metrics that are based on utilization distribution (UD) are more robust compared to the percentage area‐based overlap metrics (Fieberg & Kochanny, 2005; Kernohan et al., 2001; Millspaugh et al., 2004) which tend to have a lower discriminatory power. Applying such robust metrics especially to critically endangered species such as African white‐backed vultures may provide useful ecological knowledge regarding the spatial and social arrangement of such species in ecosystems. For example, quantifying home range overlap zones may be a useful proxy for inferring potential species interactions and encounter rates (Robert et al., 2012; Sanchez & Hudgens, 2015), (but see Vander Wal et al., 2014), which is critical for expanding our understanding of infectious disease ecology (Gangoso et al., 2009; Hoover et al., 2020), mating behaviors, territorial behavior (van Overveld, Gangoso, et al., 2020), breeding behavior (Anderson, 2004; Leepile et al., 2020; Xirouchakkis & Mylonas, 2007), sociality, and information sharing as well as the location and pulling effect of high‐quality habitats and resources (Cortés‐Avizanda et al., 2014; Jackson et al., 2008; Kane et al., 2015; Murn & Anderson, 2008). High‐quality habitats here refer to beneficial communal roosting and breeding colony zones where social information can be shared, as well as sites where food is abundant and predictable (Arkumarev et al., 2014; Cecere et al., 2018; Cortés‐Avizanda et al., 2014; Kane et al., 2015). Such sites may also provide important refuge for critically endangered vultures against several threats.

Vultures provide vital ecosystem services which are well documented (Buechley & Şekercioğlu, 2016; Margalida & Ogada, 2018; Monadjem et al., 2018; Ogada et al., 2012). Besides, they are also normally regarded as indicator species as they tend to respond quickly to environmental disturbances (Henriques et al., 2018; Pfeiffer et al., 2015; Virani et al., 2011). Albeit providing key ecological functions, their populations have continued to decline severely due to several threats such as poisoning, habitat loss, shortage of food, collision with infrastructural facilities such as power lines, as well as from persecution (Buechley & Şekercioğlu, 2016; Gilbert et al., 2006; Margalida & Ogada, 2018; Ogada et al., 2016; Safford et al., 2019; Thorley & Clutton‐Brock, 2017). It is in this context that conservation of vultures is now considered a top priority at a global scale (Buechley et al., 2019, 2021; Safford et al., 2019). One of the overarching challenges facing efforts to priorities vulture conservation is lack of spatially explicit knowledge regarding the distribution of key overlapping geographic zones and habitats supporting vulture populations in terms of food predictability as well as secure breeding and roosting sites (Buechley et al., 2019; Reading et al., 2019; Salewski, 2021). Such geographic zones are naturally not globally widespread within ecosystems, but confined to few landscapes (Margalida & Ogada, 2018). Effective wildlife management generally depends on investing maximum protection efforts to such key geographic zones (Xirouchakis et al., 2021). A clear understanding of geographic zones where African vultures tend to converge and interact within ecosystems is thus crucial for informing conservation planning (Buechley et al., 2019; Hoover et al., 2020). Such zones may also be targeted for vulture poisoning and persecutions, which further heightens the need to provide them with intensive protection.

To this end, several studies have focused on the ecology of African white‐backed vultures to inform their conservation practice. For example, Phipps et al. (2013) assessed the foraging ranges of immature white‐backed vultures, while Murn and Anderson (2008) explored the activity patterns of the same species. Spiegel et al. (2013) also compared food search efficiency between the African white‐backed vulture and the lappet‐faced vultures (*Torgos tracheliotus*), while Bamford et al. (2009) assessed the nesting preferences for the African white‐backed vultures. Such studies have undoubtedly provided essential scientific evidence to inform contemporary vulture conservation practice. However, to date, there is paucity of published literature that focus on quantifying African white‐backed vultures intra‐specific geographic overlap zones, and even for other vulture species (Reading et al., 2019). Lack of knowledge on space use sharing mechanisms among endangered species has been a major handicap for any attempts to design focused and strategic interventions meant to enhance the recovery of such vulture species (Cortés‐Avizanda et al., 2014; Kendall et al., 2012; Salewski, 2021). Several reasons have been proffered for the paucity of literature on the nature of spatial interactions among African white‐backed vultures in particular, and other vulture species in general. For instance, quantifying species interaction can be intrinsically challenging (van Overveld, Blanco, et al., 2020) as it requires spatially explicit data which is rarely available especially for wide‐ranging species such as vultures.

Recent advances in remote tracking technologies are revolutionizing wildlife studies as they provide spatially explicit high‐resolution GPS data upon which novel ecological questions underlying species space sharing mechanisms may be further investigated and detailed (Buechley et al., 2018; Miller, 2012; Urios et al., 2010). For example, the GPS system has capabilities to record the entire journey traveled by a vulture over its lifetime (Buechley et al., 2018; French et al., 2019; Hirschauer et al., 2017), which expands our ability to identify sites that are intensively utilized to enhance the survival and breeding success of the critically endangered species (Cortés‐Avizanda et al., 2014; Xirouchakkis & Mylonas, 2007). When using inconsistent conventional approaches such as citizen science to monitor animal movements and space use, such key, high‐quality habitats are often missed, making it difficult to provide them with concerted and prioritized conservation efforts. This has often been detrimental in as far as vulture conservation is concerned (van Overveld, Blanco, et al., 2020).

In this study, we make one of the first attempts to quantitatively estimate and delineate home range overlap zones for six African white‐backed vultures as a preliminary step to guide the designation of priority conservation areas for this species. To achieve this, we first, estimated the home range sizes for the six vultures and then estimated pairwise home range overlap zones between each pair (Cecere et al., 2018; Kane et al., 2015; Vander Wal et al., 2014). We also estimated dry breeding season and the wet non‐breeding season pairwise home range overlaps to test whether home range overlap trends for the two time blocks vary significantly. We used seasonality as a proxy for cyclic variation in food abundance and predictability as well as reproduction status (Reading et al., 2019; van Overveld, Gangoso, et al., 2020) and thus a major driver for dynamics in space use sharing patterns (Gil et al., 2014). Finally, we quantified home range overlap for each individual vulture between the dry breeding and the wet non‐breeding seasons to test for consistency in space use patterns which may be used as a strategy to optimize foraging efficiency. In all the cases, we assessed whether the key overlap zones were located inside protected areas or in human‐dominated landscapes which may either enhance or compromise conservation efforts.

Overall, we expected significant home range spatial overlaps between vulture pairs as they tend to breed and roost in colonies while also aggregating at ephemeral and episodic food sources enhanced by their robust information sharing strategies (Cortés‐Avizanda et al., 2014; Kane et al., 2015; Kendall et al., 2014). We also expected strong overlaps during the wet non‐breeding season when food in the form of ephemeral carcasses is scarce due to forage abundance for herbivores which reduces their mortalities compared to the dry season (Grammer, 2019; Kendall & Virani, 2012; Kendall et al., 2014). Studies have also shown African white‐backed vultures to be averse to human landscapes (Murn et al., 2016; Ogada et al., 2016; Pomeroy et al., 2015; Thiollay, 2006; Zvidzai et al., 2020) and thus we expected overlap zones to be concentrated within protected areas. Understanding such dynamics in space use may provide deep and useful insights for spatial conservation planning of African white‐backed vultures to support the recovery of their rapidly declining populations.

## 2 MATERIALS AND METHODS

### 2.1 Study area

**FIGURE 1:**
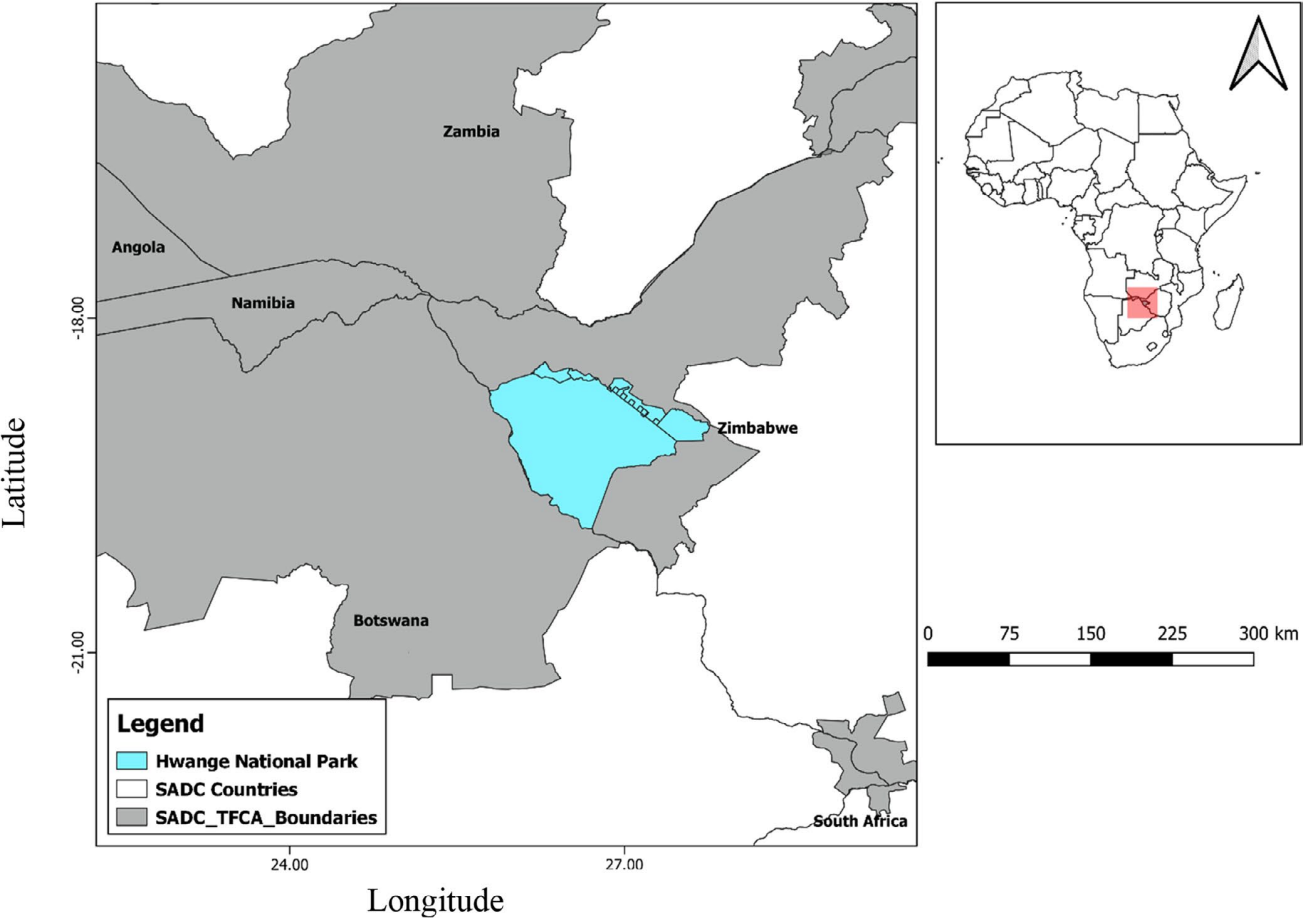
Location of the Hwange National Park within the Kavango–Zambezi Transfrontier Conservation Area

The study was conducted in the Hwange conservation area, which is part of the extensive Kavango–Zambezi conservation area (KAZA). KAZA stretches from Northwestern Zimbabwe into Zambia, Botswana, Namibia, and Democratic Republic of Congo (DRC) (Figure 1). The Hwange conservation area is largely within the savanna biome with temperatures ranging between 20 and 30°C (Chamaille'‐James et al., 2007; Mpakairi et al., 2020). The area receives seasonal rainfall averaging between 550 and 650 mm which falls normally between November and April. Hwange conservation area is dominated by Kalahari sands and as such adapted vegetation species such as *Baikiaea plurijuga*, *Colophospermum mopane* and *Terminalia* spp. are widespread in the area (Mpakairi et al., 2020; Valeix et al., 2007). An extensive network of key conservation landscapes such as national parks, private conservancies, biological corridors, buffer zones, and safari areas form part of the Hwange ecosystem. The Hwange ecosystem is considered an important bird area and is one of the few habitats where critically endangered African white‐backed vultures (*Gyps africanus*) still breed and range naturally in large numbers (BirdLife International, 2018; Mundy, 1992).

### 2.2 Vulture capturing and tagging

Detailed information about the capture process, vulture characteristics, and tracking periods is provided in Zvidzai et al. (2020). Here, we only provide brief information. Six African white‐backed vultures were captured at two different sites within Hwange National Park using a modified cannon net projection system (Bamford, Monadjem, Diekmann et al., 2009; Young & Bruce, 2002). We used a goat carcass as a bait. Vultures 1, 2, and 3 were captured at the same site while Vultures 4, 5, and 6 were captured at another site. The capturing sites were about 85 km apart. We did not determine the sex of the captured vultures because we did not have any ecological reasons to expect any variations in space use patterns between male and female white‐backed vultures. In fact, several previous studies in Africa and elsewhere did not find any significant differences in ranging patterns between male and female vultures of other species (Bamford et al., 2007; García‐Ripollés et al., 2011; Kane et al., 2016; Krüger et al., 2014; Margalida & Ogada, 2018; Reading et al., 2019).

After capture, the vultures were fitted with solar‐charged 3G CTT™ 1000 Series BT3 GPS tracking devices which were harnessed at the back using Teflon ribbons to secure the backpack (Curk et al., 2020; Kendall & Virani, 2012; Pfeiffer et al., 2015; Price‐Rees & Shine, 2011). The capturing and tagging processes were approved and monitored by Zimbabwe Parks and Wildlife Management Authority under Permit No. 23(1) (C) (II) 23/2016. The GPS devices were programmed to record a fix after every 15 min starting from 05:00 a.m. to 5:00 p.m. This is mainly because vultures are only active during the day and as such the devices were programmed to be active during the day and inactive during the night to save battery life. Before data were analyzed, all erroneous fixes were removed (Walter et al., 2011).

### 2.3 Home range estimation

To get best possible results from home range and home range overlap estimates, data were tested if it met the range residency criteria. This is because any data which do not show evidence of range residency is not suitable for home range analysis (Fleming et al., 2015). In this regard, Fleming & Calabrese (2017) recommends visualization of the variogram to validate if data meet the range residency assumption for it to be suitable for home range and home range overlap analysis. Under range residency conditions, the variogram should indicate a clear saturation of home range at some point in time (Calabrese et al., 2016). Once range residency status for each vulture was established, selection of candidate movement model to estimate home range was then employed to identify the best model for the data (Calabrese et al., 2016). Here, we considered two candidate models, that is, Ornstein–Uhlenbeck (OU) and Ornstein–Uhlenbeck–F (OUF) (Fleming & Calabrese, 2017; Fleming et al., 2014).

To estimate home ranges for the vultures, we used the non‐parametric autocorrelated kernel density estimator (AKDE) at the 95% isopleth (Fleming et al., 2015). The rigorous AKDE was developed largely to remedy the shortfalls associated with conventional home range estimators such as the kernel density estimator (KDE) whose assumptions are not compatible with modern highly autocorrelated GPS tracking data (Fleming et al., 2015). The AKDE applied here has capabilities to explicitly correct for biases due to autocorrelation in movement data as well as biases due to under‐ or over‐sampling (Fleming et al., 2015). These are norms usually associated with modern, high‐resolution GPS technologies. Autocorrelation in the tracking data is explicitly accounted for by using information theoretic methods to select and fit the most appropriate candidate model for the data, and then based on the same selected autocorrelation model, apply the optimized Gaussian reference smoothing function for the KDE (Fleming et al., 2015, Fleming & Calabrese, 2017).

### 2.4 Home range overlap estimation

To estimate home range overlap, data for the six vultures were first arranged in a pairwise matrix forming dyads. A dyad was defined as a pair of specific individuals, regardless of whether there was any spatial overlap in their home ranges. For example, Vulture 1 and Vulture 2 would form a possible dyad which we labeled dyad 1, while Vulture 1 and Vulture 3 would form another dyad which we labeled dyad 2. Based on this approach, 15 possible vulture dyads were established for this study (Table 1).

**TABLE 1:** Possible dyads for the six vultures. All the six vultures were paired against each other resulting in 15 possible dyads

	V1	V2	V3	V4	V5	V6
V1		V1–V2	V1–V3	V1–V4	V1–V5	V1–V6
V2			V2–V3	V2–V4	V2–V5	V2–V6
V3				V3–V4	V3–V5	V3–V6
V4					V4–V5	V4–V6
V5						V5–V6
V6						

To compute home range and home range overlaps, we used the AKDE and the robust Bhattacharyya coefficient (BC) (Bhattacharyya, 1943; Fieberg & Kochanny, 2005; Winner et al., 2018). The BC quantifies similarities between a pair of utilization distributions (UDs) normally referred to as home range overlap, with values ranging between 0 (complete segregation of home ranges) and 1 (perfectly overlapping home ranges or identical UDs). Unlike the percentage‐based home range overlap, the BC index is modeled based on species utilization distributions and thus quantifies similarities between two probability distributions (UDs) (Fieberg & Kochanny, 2005). Thus, it can provide a more accurate estimate of pairwise species home range overlap (French et al., 2019). Furthermore, the BC, as applied within the AKDE environment, is one of the first overlap metrics which can estimate confidence intervals to quantify the level of uncertainty in estimated home range and home range overlaps (Winner et al., 2018).

We quantified vulture pairwise home range overlap estimates at two levels that is based on pooled data and seasonal data. For the pooled data, home range overlap for each dyad was quantified based on all the tracking data. At the seasonal level, home range overlap for each dyad was computed for the dry breeding season as well as the wet non‐breeding season after which they were tested if they were significantly different. We also quantified home range overlap for each vulture between the wet non‐breeding season and the dry breeding season to test for consistency in space use patterns. The dry breeding season in this case was estimated to start from May until October while the wet non‐breeding season starts from November until April (Mundy, 1992).

All the home range and home range overlap workflows were performed through the continuous‐time movement modeling (ctmmweb), a web‐based and R Shiny‐based (Wickham et al., 2019) graphic user interface (GUI) within the R environment following Dong et al. (2017) and Calabrese et al. (2021).

## 3 RESULTS

**FIGURE 2:**
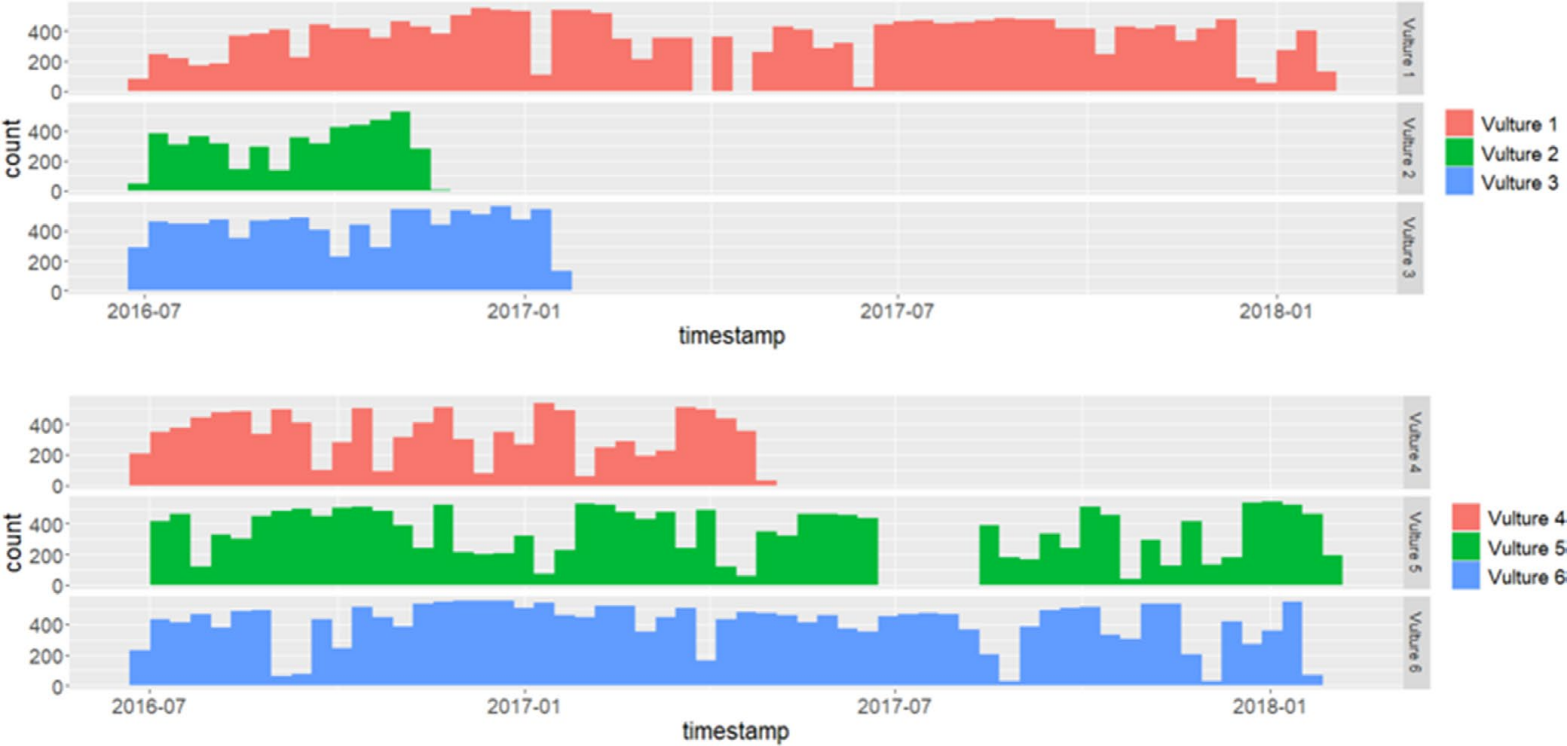
Tracking period for the six vultures and the number of monthly GPS fixes from June 2016 to January 2018. The *X*‐axis shows the tracking period by year and month (e.g., 2016–07 designates July 2016) and the *Y*‐axis shows the number of GPS fixes per month for each of the six individual vultures. Short bars show fewer fixes for that month while longer bars show that more fixes were collected for that month

Figure 2 shows the tracking period for each vulture and the number of fixes collected per month and then per year. Vultures 1, 5, and 6 were tracked the longest, from June 2016 to January 2018. Vulture 2 had the shortest tracking period from June 20016 to November 2016 (Figure 2).

**FIGURE 3:**
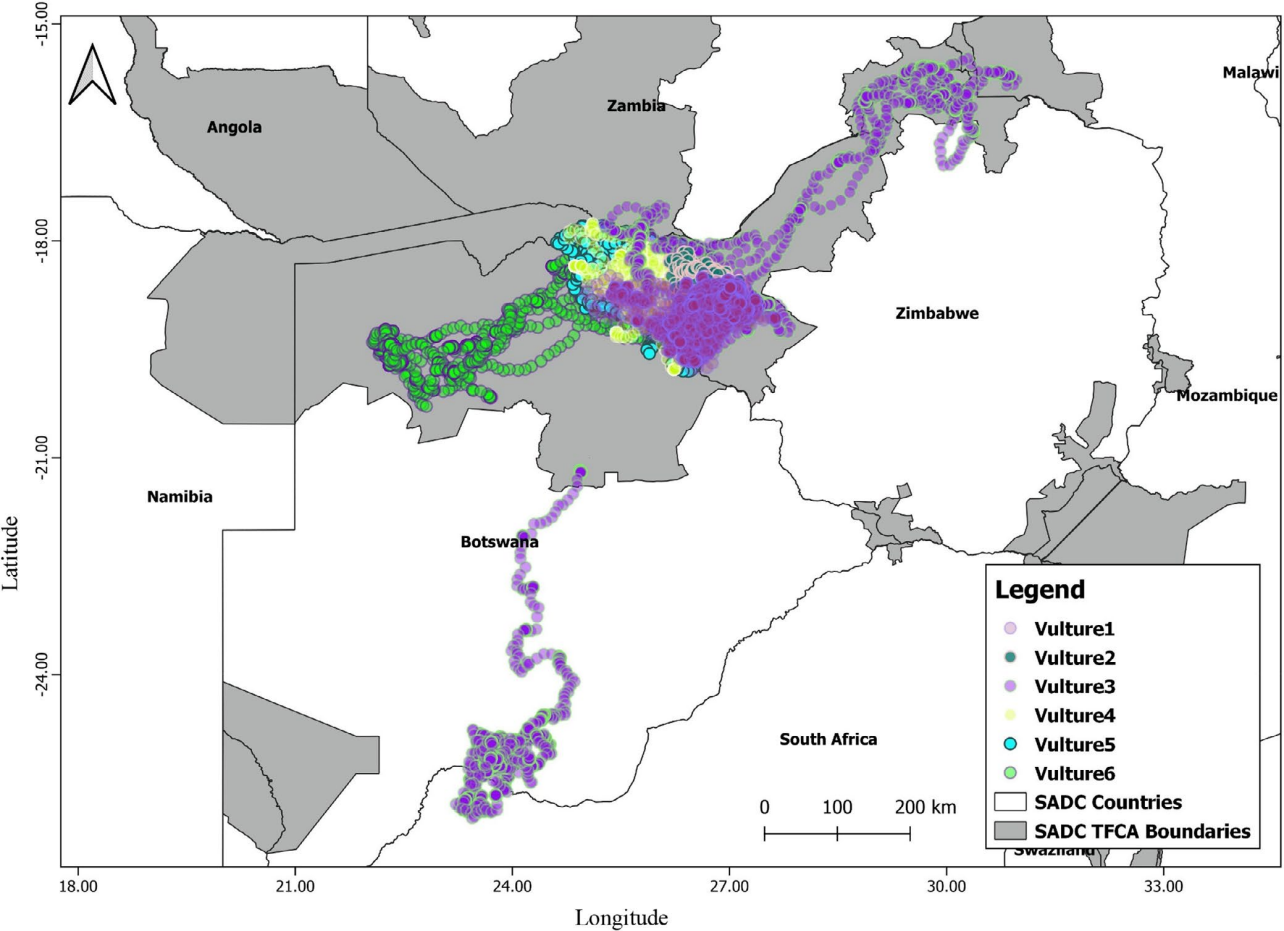
Movement patterns for the six vultures showing Vulture 3 with the longest geographic range. Five vultures had their movements confined to conservation areas while only Vulture 3 had the longest stretch of movement outside conservation areas between Botswana and South Africa

The movement patterns for the six vultures for the entire tracking period varied. Vultures 1, 2, 4, 5, and 6 movements were largely confined around the capture site within Hwange National Park. Vultures 3 had the widest geographic range away from the capture site (Figure 3). Vulture 6 had some movements into the Chobe National Park during the wet non‐breeding season (Figure 3).

### 3.1 Home range model selection

**FIGURE 4:**
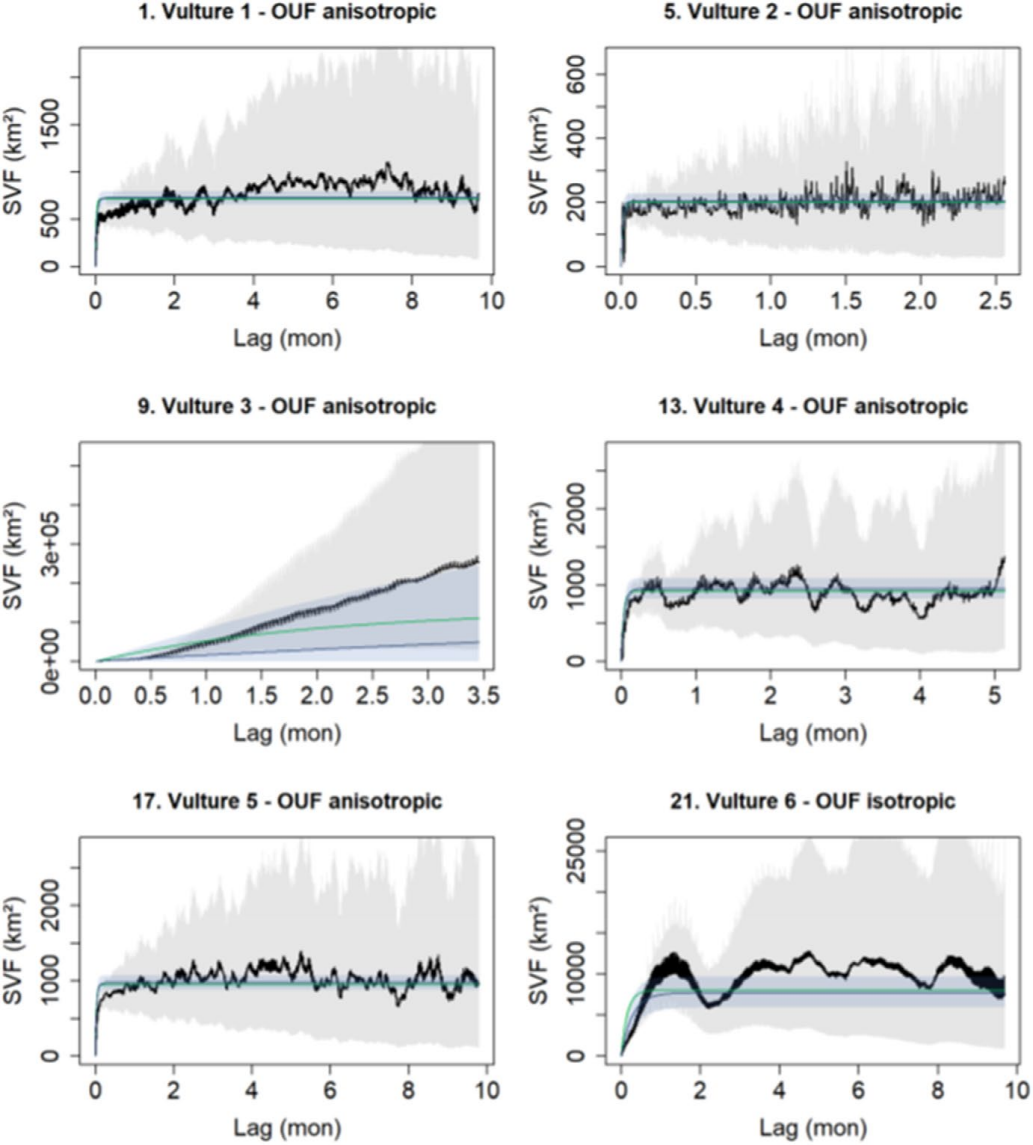
Home range autocorrelation model selection for the six vultures at 15 min fix interval. Vulture 3 shows that its home range does not reach saturation point and, as a result, it was not included in the analysis

The superior home range model for each of the six vultures based on pooled data is shown in Figure 4. The OUF anisotropic model was the superior model for all the vultures except Vulture 6 which had best fit on OUF isotropic model (AICc = 0.00).

### 3.2 Home range sizes and home range overlaps

Vulture 3 did not meet the range residency criteria. As such it was not included in home range analysis, thus reducing our dyads from 15 to 10 dyads. Vulture 4 had the smallest home range size of 3 532.9 km^2^ (CI 3108.9–3 983.6) while Vulture 6 had the largest home range size of 75 543.8 km^2^ (CI 58 400.9–94 859.3). The mean home range size for the five vultures was 24 768.7 km^2^. For the wet non‐breeding season, Vulture 6 again had the most extensive home range size of 176 910.6 km^2^ (CI 102 718.9–270 939.9) while Vulture 1 had the smallest home range size of 8 413.9 (CI 7 107.5–9 828.9). Home range sizes for the wet non‐breeding season were larger than home range sizes for the dry breeding season. Because Vulture 6 had the overall largest home range estimate, home ranges for all other four vultures were entirely nested within the bigger Vulture 6 home range.

Regarding home range spatial overlaps, there was no evidence of complete home range spatial segregation among the five vultures since some degree of overlap was recorded across all dyads. Vultures 1 and 5 had the weakest estimated home range overlap of 0.21 (CI 0.18–0.25) for the pooled data while Vultures 4 and 5 had an almost perfect home range overlap of 0.97 (CI 0.96–0.98) (Table 2).

**TABLE 2:** Home range overlaps for the 10 dyads based on pooled data at 95% confidence level

Dyad	V1	V2	95% Established HR Overlap	95% CL
1	Vulture 1	Vulture 2	0.44	0.40–0.48
2	Vulture 1	Vulture 4	0.31	0.25–0.37
3	Vulture 1	Vulture 5	0.21	0.18–0.25
4	Vulture 1	Vulture 6	0.37	0.30–0.45
5	Vulture 2	Vulture 4	0.29	0.24–0.34
6	Vulture 2	Vulture 5	0.23	0.20–0.27
7	Vulture 2	Vulture 6	0.32	0.26–0.39
8	Vulture 4	Vulture 5	0.97	0.96–0.98
9	Vulture 4	Vulture 6	0.77	0.68–0.86
10	Vulture 5	Vulture 6	0.80	0.71–0.88

**FIGURE 5:**
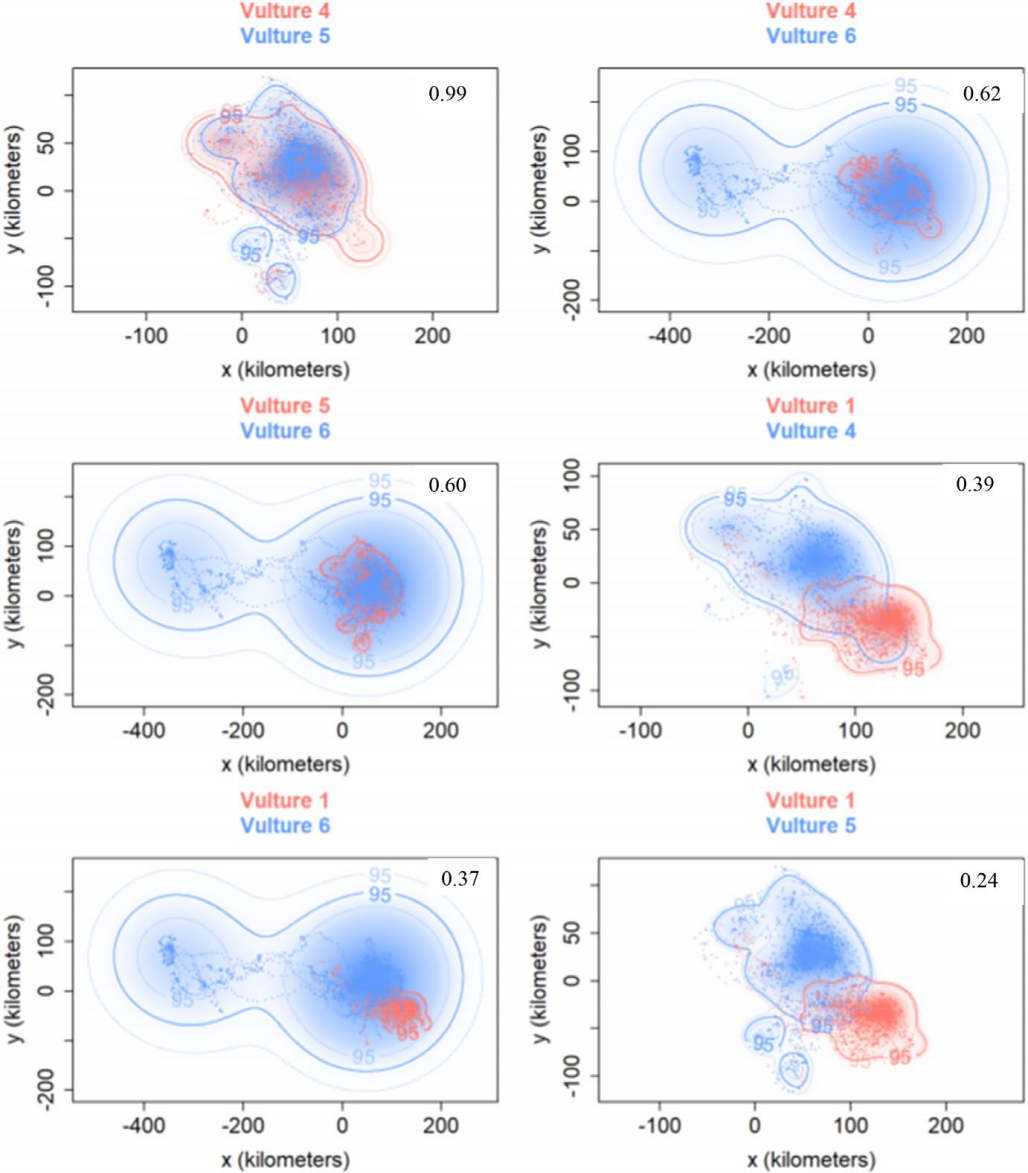
Wet non‐breeding season home range overlaps for the four African white‐backed vultures at 95% CI. Estimated home range overlap values for each dyad are inside the plots. Vulture 2 was not considered here because it was tracked for the dry breeding season only

For the wet non‐breeding season, Vultures 1 and 5 had the weakest home range overlap of 0.24 (CI 0.18–0.3) while Vultures 4 and 5 again had the strongest, almost perfect overlap of 0.99 (CI 0.96–1.00) (Figure 5).

**FIGURE 6:**
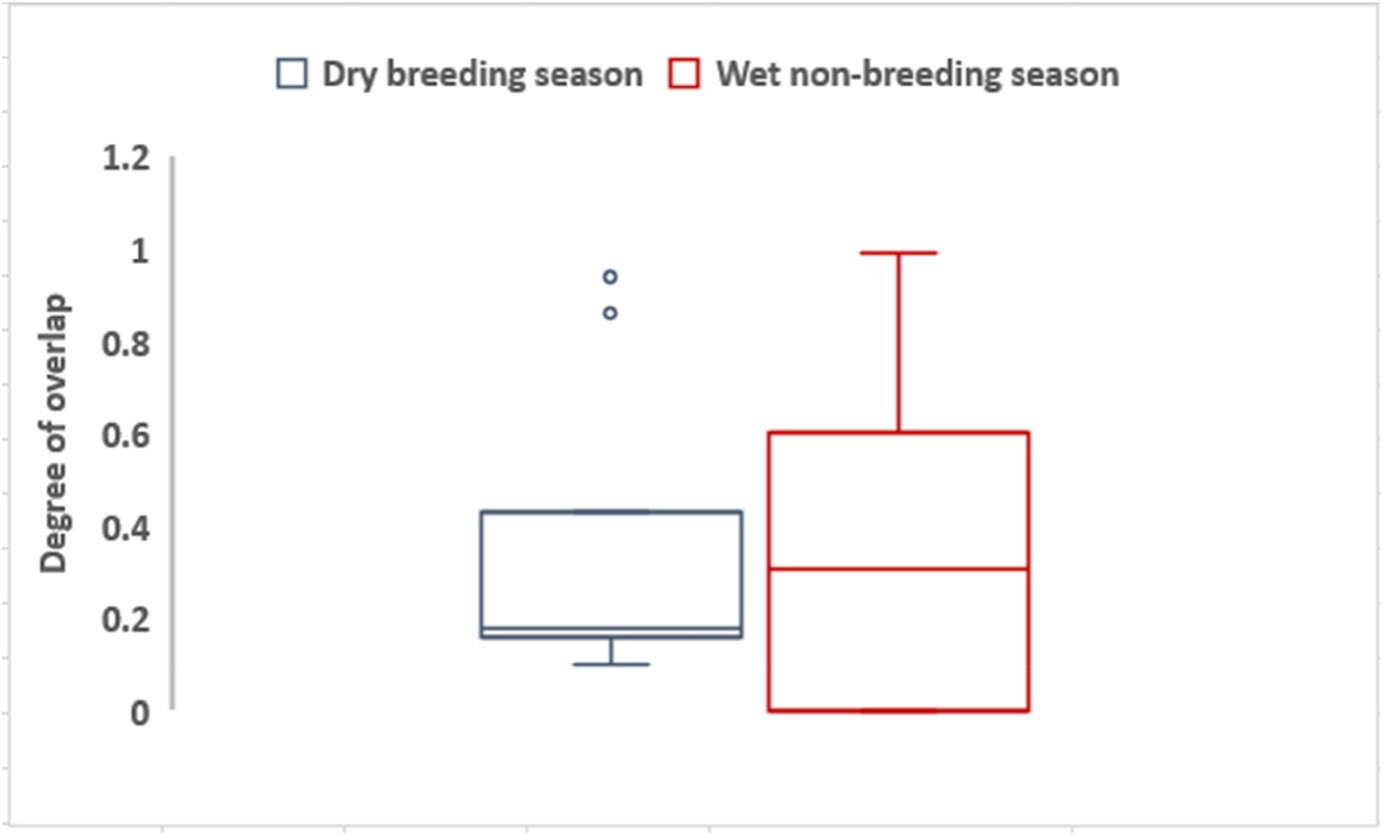
Box and whisker plots showing the distribution of home range overlaps for the dry breeding season and the wet non‐breeding season

Average home range overlap for the wet non‐breeding season was 0.32 ± 0.23 while the average for the dry breeding season was 0.34 ± 0.28 which shows a marginally small difference (Figure 6).

### 3.3 Individual birds UD overlap between dry breeding and wet non‐breeding season

**FIGURE 7:**
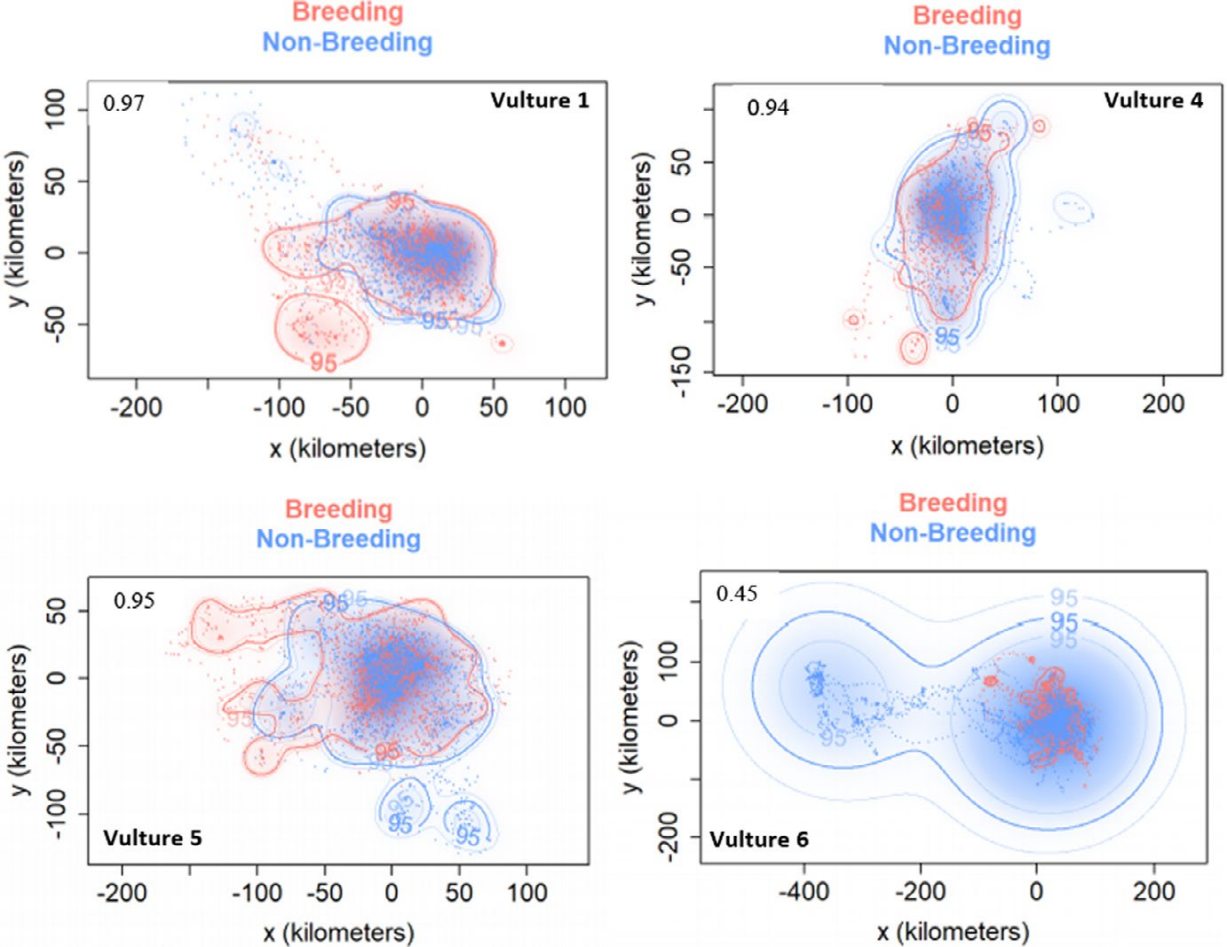
Between‐season (dry breeding and wet non‐breeding) home range overlaps for four individual vultures at 95% CI. Estimated home range overlap values are inserted inside each plot. Vulture 2 is not included here because it was only tracked for one season

Vulture 1 dry breeding and wet non‐breeding season home ranges had the strongest spatial overlap of 0.97%, (CI 0.94–0.99). Three other vultures (Vulture 1, 4, and 5) also showed consistency in space use as their breeding and non‐breeding season UDs overlapped by over 0.90. However, Vulture 6 had the weakest consistency in space use between seasons as suggested by the low overlap of 0.45 (CI 0.34–0.57) (Figure 7).

## 4 DISCUSSION

Our study provides some first insights for understanding home range spatial overlaps or segregation among the critically endangered African white‐backed vultures. Although our findings are based on a small sample size (*n* = 5), which limits the generalization of our study, results from this study provide a first key step for informing prioritization of conservation actions targeting wide‐ranging and endangered species where conservation resources are limited. Besides, our study may also provide the basis upon which other vital downstream ecological questions regarding social evolution, information sharing, and disease ecology can be investigated (Signer & Fieberg, 2021) to expand our knowledge on the spatial ecology of critically endangered vulture species.

One key finding of our study was that core overlap zones for the white backed vultures are mainly concentrated within the Hwange National Park which is consistent with one of our expectations. This is also consistent with previous studies which demonstrated that white‐backed vultures tend to show strong site fidelity to protected areas, while avoiding human‐dominated landscapes (Murn et al., 2016; Ogada et al., 2016; Pomeroy et al., 2015; Thiollay, 2006). It was therefore not surprising for home range overlap zones to be entirely within protected areas. This may be explained by consistent and reliable carcass abundance in protected areas due to starvation, diseases, and sometimes poaching incidents, compared to human‐dominated landscapes where improved livestock management practices has resulted in drastic reductions in mortalities of domesticated animals (Kendall et al., 2014; Pomeroy et al., 2015; Thiollay, 2006). Our preliminary results thus reaffirm the critical contribution of protected areas to the conservation of critically endangered African vulture species (Murn et al., 2016; Salewski, 2021; Xirouchakkis & Mylonas, 2007). Our results are, however, contrary to findings from other studies which showed that some vulture species tend to prefer human‐dominated landscapes compared to protected areas, thus posing several conservation challenges to wildlife managers (Buechley et al., 2021; Henriques et al., 2018; Phipps et al., 2013). Such vultures could probably be attracted by sporadic food sources found in human landscapes (Murn & Anderson, 2008; Phipps et al., 2013). When key ranging zones are concentrated outside protected areas, vultures are exposed to several threats such as poisoning, collisions with human infrastructure, as well as conflicts with humans (Buechley et al., 2021; Phipps et al., 2013).

Home range sizes estimated in this study (mean 24 768.7 km^2^) are substantially smaller compared to those found by Phipps et al. (2013) (mean GCRs 56,683.6 ± 9,210 km^2^ and mean KDE 33,922.3 km^2^) who studied immature African white‐backed vultures using GPS data. Bamford et al. (2007) also demonstrated extensive home ranges for immature Cape vultures (*Gyps coprotheres)* (MCP 482,276 km^2^). This may not be surprising since immature vultures normally range widely in an attempt to establish favorable home ranges (Hirschauer et al., 2017). Nomadic tendencies for immature vultures may also be inferred as deliberate strategies to find food as well as unrelated breeding partners to avoid inbreeding within the population (Hirschauer et al., 2017; Monadjem et al., 2013; Phipps et al., 2013). However, mean home range sizes in this study are much comparable to those for mature Cape vultures (MCP 21 320 km^2^) (Bamford et al., 2007), but are larger than home range estimates for griffon vulture *Gyps fulvus* (Xirouchakis et al., 2021). We suspect that variations in home range sizes could be explained by different species traits, as well as different methods and fix intervals applied to estimate home ranges. Previous studies have demonstrated that MCP and KDE methods tend to overestimate home range sizes (Fleming & Calabrese, 2017; Fleming et al., 2015; Worton, 1995) while other studies have also shown that home range estimates from different GPS fix intervals tend to differ significantly (Kolodzinski et al., 2010; Mills et al., 2006; Zvidzai et al., 2020). Here, based on a robust algorithm, the AKDE, which even accounts for autocorrelation (Fleming & Calabrese, 2017; Fleming et al., 2015; Winner et al., 2018), we make a claim that home range estimates in our study could be more precise compared to estimates from previous studies. A study by Winner et al. (2018) indeed demonstrated that AKDE is much more accurate when compared to other home range estimators. Spatial conservation planning requires precise information on space use patterns. Accurate home range estimates are therefore indispensable for effective conservation of critically endangered vulture species.

We expected strong home range overlaps among the five vultures but contrary to our expectation, the five vultures showed lower average UD overlap of 0.38 ± 0.26 based on pooled data and 0.34 ± 0.28 based on dry breeding season data. For the wet non‐breeding season, the average UD overlap was 0.32 ± 0.23. Our expectations for intensive home range overlaps were based on two assumptions. First, African white‐backed vultures range extensively and tend to establish social networks to enhance their foraging success (Monadjem et al., 2018; Phipps et al., 2013). Second, several studies have identified vultures as highly gregarious species which breed and roost in loose colonies (Bamford et al., 2009; Harel et al., 2017; Mundy, 1992; van Overveld, Gangoso, et al., 2020). Such behavior may support intensive spatial overlaps in home ranges. We are not sure why such weak spatial overlaps were revealed in our study, but we speculate that this could be evidence of complex social structuring among scavenger populations. We also suspect that the small sample size in our study may have contributed to the weak spatial overlap metrics reported here. For example, some tagged vultures were probably having strong spatial overlaps with other vultures which were not part of our study.

Although Gil et al. (2014) studied home range overlaps for bearded vultures *Gypaetus barbatus*, they did not quantify home range overlaps, making it difficult to compare their study with ours. Previous studies that have classified vultures as gregarious and social species based their findings on observation data collected at carcasses (Kendall et al., 2012; van Overveld, Gangoso, et al., 2020). Some of such studies were also based on citizen science where data collection is not systematic (Monadjem et al., 2014, 2018; Thorley & Clutton‐Brock, 2017). Our results are based on high‐resolution, spatially explicit data collected systematically over a reasonably long period of time. As such, it is reasonable to expect different results from such disparate study approaches.

Vultures 1 and 2 were captured and tagged at the same site. Thus intuitively, we expected some social ties to exist between them. However, our results suggested that the two vultures exhibited weak spatial overlaps across all scales. Some previous studies have observed that vultures in general tend to exhibit complex social grouping dynamics (Grammer, 2019; Kappeler et al., 2019; van Overveld, Blanco, et al., 2020) probably as a strategy to counter the sporadic and ephemeral nature of their food sources. For example, while other studies have suggested communal foraging among vultures, several cases of solitary foraging have been observed (Harel et al., 2017; Kappeler et al., 2019). Several cases were also observed where vultures moved in small, dispersed groups while large aggregations were only common around carcasses as well as at communal roosts or resting areas (Kendall et al., 2012; van Overveld, Gangoso, et al., 2020). This could possibly explain the weak overlaps in this study. Besides, vulture species usually form social networks when foraging (Cortés‐Avizanda et al., 2014; Pinter‐Wollman et al., 2013). As a result, they tend to be dispersed over a wide area within their perceptual range (Harel et al., 2017) to enhance their foraging efficiency through information sharing (Spiegel et al., 2013). Due to their telescopic eye sight, they rely on cues from other vultures once a carcass has been opportunistically identified, and this will prompt them to follow swiftly those which may have information on where food could have been located (Harel et al., 2017). The wide dispersal when foraging could possibly explain the weak home range overlaps suggested in this study. A study by Xirouchakis et al. (2021) also demonstrated weaker home range overlaps among griffon vultures *Gyps fulvus*, which corroborates our results for Vultures 1 and 2.

On the other hand, Vultures 4, 5, and 6 which were also captured and tagged at the same site exhibited strong spatial home range overlaps. The three vultures consistently showed intensive home range overlaps at all levels, which is based on pooled data as well as across seasons. Based on this observation, we can infer the existence of social ties among the three vultures (Vultures 4, 5, and 6) which could possibly explain the consistent spatial attraction for their home ranges. Robert et al. (2012) found a strong correlation between home range overlap and species encounter rates which could have implications for the spread of diseases which have been cited as one of the factors contributing to vulture declines (Gangoso et al., 2009). The strong spatial overlaps could suggest frequent and intensive contacts among the vultures, which could suggest shared key breeding, roosting, and feeding zones. Such zones may require preferential conservation attention. A study by Vander Wal et al. (2014) also corroborated a clear relationship between home range overlap and encounter rates. However, the study cautioned against the general application of home range overlaps as a proxy for species encounter rates and argued that besides being site specific, the relationship is much complex as it may also depend on other factors such as species population density, familial ties among the species, as well as external ecological factors (Vander Wal et al., 2014). However, for gregarious species such as African vultures, which feed in groups and roost in colonies, encounter rates may be more strongly correlated with home range overlap than for non‐social species.

Seasonality is often cited as a surrogate for cyclic food availability, in which case it is purported to regulate space use sharing patterns among vulture populations and other species (Arkumarev et al., 2014; Kendall et al., 2014; van Overveld, Gangoso, et al., 2020; Reading et al., 2019). For example, food in the form of carcasses is freely distributed during the dry breeding season, while the wet non‐breeding season is normally characterized by low carcass densities (Kendall et al., 2014, Grammer, 2019, Estevinho Santos Faustino, 2020). Thus, the concentration at carcasses should be higher when more food is freely distributed, and predictable as during the dry breeding season (Møller & Laursen, 2019; van Overveld, Gangoso, et al., 2020). This would naturally result in increased home range overlaps. Previous studies have shown that raptors gregarious tendencies at carcasses were more prevalent in areas of high carcass density (Hill et al., 2018; Kendall et al., 2012; Zuberogoitia et al., 2010). However, our results showed small differences in home range overlaps between the dry breeding season and the wet non‐breeding season. This finding was surprising since we expected huge differences in between‐season home range overlaps. However, the weak overlaps for the wet non‐breeding season were largely expected. The wet non‐breeding season (November to April) is usually characterized by low and patchy carcass densities (Kendall et al., 2012, 2014), thus vultures are expected to range widely to access sporadic food sources, thus less aggregation (Kane et al., 2015; Zuberogoitia et al., 2010). When food is scarce and patchy, the tendency is for vultures to be dispersed over large areas compared to when food is readily available (Harel et al., 2017). This may translate to weak home range spatial overlaps between vulture pairs suggested during the wet non‐breeding season in this study. Future studies based on larger sample size are, however, required to further test the effect of seasonality on home range spatial overlaps for African vultures.

One of the key objectives of this study was to test how individual vultures were consistent in space use between the dry breeding season and the wet non‐breeding season. We tested this by quantifying home range overlaps for each vulture for the two seasons. Our results suggest higher between‐season home range overlaps for Vultures 1, 4, and 5, while Vulture 6 had a weaker home range spatial overlap between seasons. We expected lower between‐season home range overlaps perhaps since the dynamics of food abundance and predictability is largely driven by seasonality (van Overveld, Gangoso, et al., 2020). Contrary to our expectations, our results demonstrated that vultures in this study are probably philopatric within the Hwange conservation area, but with occasional forays to Chobe National Park of Botswana. Previous studies agree with our findings as they also demonstrated that *Gyps* vultures tend to show strong site fidelity across seasons (Monadjem et al., 2016; Murn & Anderson, 2008; Pfeiffer et al., 2015; Prakash et al., 2007; Zvidzai et al., 2020). Other studies have also shown that mature vultures are normally strongly attached to their key breeding and roosting zones, and in most cases only traveling short distances away from their nests (Gil et al., 2014; Monadjem et al., 2018). This could suggest a situation where the vultures have well established themselves and acquired knowledge on the spatial and temporal distribution of high‐quality habitats which guarantees higher foraging efficiency and reproductive success as well as refugia. Thus, such hotspot zones may need to be afforded selective conservation status (Harel et al., 2017; Margalida & Ogada, 2018; Santangeli et al., 2019) to enhance the recovery of white‐backed vulture populations.

Our study provides some of the first and vital empirical evidence on utilization distributions and intra‐specific home range overlaps patterns for white‐backed vulture species. In that context, home range overlap estimates reported here are important for spatial conservation planning, especially with regards to critically endangered African vultures. The findings provide new insights which can inform our understanding of space use sharing among vulture populations and how such spatial arrangements can drive other ecological and social processes such as social evolution, information sharing, and the disease ecology (Gangoso et al., 2009; Hoover et al., 2020; Leepile et al., 2020; van Overveld, Blanco, et al., 2020). However, because the sample size of our study is small, caution should be taken when interpreting our findings.

To this end, results of our study, although preliminary, have critical conservation implications for the imperiled African white‐backed vulture populations, which naturally range widely, and sometimes crossing international boundaries (Phipps et al., 2013, Hirschauer et al., 2017, Estevinho Santos Faustino, 2020). Such a scenario may present several conservation challenges (Buechley et al., 2018; Margalida & Ogada, 2018). Knowledge of key utilization distributions and overlap zones which may correspond to high‐quality geographic zones such as breeding colonies, communal roosting sites, water, and carcasses is thus vital as it allows focusing conservation efforts on much smaller but essential zones. This may then justify the deployment of constrained conservation resources to such strategic zones. In fact, a study by Loveridge et al. (2020) showed that focusing conservation efforts on key vulture breeding colonies resulted in substantial breeding and fledging success (Bamford, Monadjem, & Hardy, 2009).

## CONFLICT OF INTEREST

The authors declare no conflict of interest.

## AUTHOR CONTRIBUTIONS

**Mark Zvidzai:** Conceptualization (lead); Formal analysis (lead); Investigation (lead); Methodology (lead); Project administration (supporting); Resources (supporting); Software (equal); Validation (equal); Visualization (equal); Writing – original draft (lead); Writing – review & editing (lead). **Fadzai Michelle Zengeya:** Conceptualization (supporting); Formal analysis (supporting); Investigation (supporting); Methodology (supporting); Resources (equal); Supervision (lead); Validation (supporting); Writing – original draft (supporting); Writing – review & editing (supporting). **Mhosisi Masocha:** Conceptualization (supporting); Formal analysis (supporting); Methodology (supporting); Supervision (lead); Validation (supporting); Visualization (supporting); Writing – original draft (supporting); Writing – review & editing (supporting). **Henry Ndaimani:** Data curation (supporting); Formal analysis (supporting); Investigation (supporting); Project administration (supporting); Software (lead); Validation (supporting); Writing – original draft (supporting); Writing – review & editing (supporting). **Amon Murwira:** Conceptualization (supporting); Funding acquisition (lead); Project administration (equal); Resources (lead); Supervision (supporting); Writing – original draft (supporting); Writing – review & editing (supporting).

## Data Availability

The GPS location data used in this study are sensitive as it pertains to a critically endangered species. Making the data publicly available may further expose the species to dangers of poaching and poisoning.
